# Motivating young adults to connect with nature for stress relief: A study in Taiwan during the COVID-19 pandemic

**DOI:** 10.3389/fpsyt.2022.922107

**Published:** 2022-09-06

**Authors:** Yin-Yan Yeung, Chia-Pin Yu

**Affiliations:** ^1^School of Forestry and Resource Conservation, National Taiwan University, Taipei, Taiwan; ^2^The Experimental Forest, National Taiwan University, Nantou, Taiwan

**Keywords:** nature, virtual nature, motivational enhancement, young adults, pandemics, connectedness to nature, perceived stress

## Abstract

It is known exposure to and connectedness with nature is beneficial for psychological wellbeing and stress relief. However, many factors hinder people, including young adults, from utilizing natural resources for healing. The present study indicates using a motivational enhancement approach and additional motivational elements in public messaging to address ambivalence toward nature exposure successfully results in favorable impacts on belief, intention, recall of positive nature elements, and perceived stress. Because this study coincided with the development of the COVID-19 pandemic in Taiwan, it offers a valuable opportunity for exploring how effective the motivational indicators were at different stages of the pandemic, as well as how connectedness to nature can explain perceived stress. In measuring motivation, we captured the essential elements of mobilizing young adults to connect with nature while also exploring potential expansion of behavioral indicators. We discuss how to foster inspiration during a pandemic to enhance connectedness to nature.

## Introduction

During the pandemics, social distancing under the lockdown measures, anxiety toward being infected as well as the uncertainty in future planning can put young adults, who are most vigor for social activities and passionate for making roadmap for their life, in stress. Actually, the current generation of young adults may not be experiencing the most enjoyable period of their lives and may not be in their healthiest psychological state, which runs contrary to what is generally expected for people in this age group. From 2008–2017, serious psychological distress in the past month and suicide-related outcomes in the past year increased for the 18–25 age group (1). More than a quarter of a sampled university student population in Taiwan reported a poor mental health status, and more than 60% of the respondents had experienced stressful events in the past year (2). A study of college students spanning nine countries reported 64.3% of respondents had experienced stress related to their health (3), implying that, for young adults, health concerns can be a trigger for stress. With the outbreak of the pandemic, college students are expected to experience increased stress levels as the general population has. It is worth exploring effective methods that improve the mental health of young adults. Using exposure to nature or connection with nature is one possible option.

Empirical evidence confirms younger adults (of age 18–34), when compared with older adults (of age 35–54, and 55 or above), fared the worst during the COVID-19 pandemic with regards to symptoms of depression, stress, and anxiety (4). Most college students (85%) suffered psychological impacts during the pandemic (5), and this was also true for Chinese young adults. Analysis of 17, 865 online social platform users with a median age of 33 found that symptoms of depression and anxiety increased after the onset of COVID-19, while life satisfaction and positive emotions decreased (6). The psychological impact on college students has been reported (7), with nearly 10% of students having developed or maintained mental health problems during the pandemic (8).

A systematic review revealed an unfortunate outcome, that many young people are reluctant to seek help from formal mental health services (9). Visiting nature, which is a leisure activity, has a positive impact on mental health (10) by decreasing depression, anxiety (11), and stress (12, 13). As such, visiting nature may be an effective treatment approach for young people, whose health and psychological wellbeing are associated with autonomy needs gratification (14, 15). For cognitive and emotional benefits, a 50-min walk in nature elicits the desired effects (16). A review of evidence revealed biological markers of stress, namely cortisol, decrease in natural environments (17), and a nature experience as brief as 20 to 30 min can result in positive effects (18).

Despite the known benefits that result from exposure to nature, growing older (19), urbanization (20) and the increased use of electronic screen technology (21) have deterred people from spending time outdoors and has lowered their eagerness to connect with nature. During the pandemic, continuous exposure to nature could improve the health of the participant. Views of nature are associated with lower levels of somatization (22), not to mention calmness, stress relief, and anxiety reduction in young people (23). Young people have reported wanting to spend more time in nature but have encountered barriers to doing so (23). In view of the increased stress experienced by young people during the pandemic and their ambivalence toward visiting nature, as well as the evidence of the benefits of nature exposure on mental health and stress reduction, it is prudent to explore an evidence-based approach for motivating young people to connect with nature during stressful periods.

Self-determination theory (SDT) (24, 25) depicts how autonomous motivation and behavioral change can be achieved by addressing psychological needs of autonomy (i.e., self-driven), competence (i.e., feeling of mastery and self-efficacy), and relatedness (i.e., connections not restricted to one person but with a wider community as well). On the other hand, outcome expectancy (26) has successfully been applied in promoting health behaviors, such as physical exercise (27), drinking/smoking/weight control (28), and medical adherence behavior (29). Given that overly positive expectations can in turn lead to negative treatment outcomes (30), setting realistic expectations is of the utmost importance.

Motivational Interviewing (M.I.) (31) is a “client–centered, directive method for enhancing intrinsic motivation to change by exploring and resolving ambivalence” [(31), p. 25]. While direct persuasion may not be effective and may increase resistance, M.I. advocates recognition of an individual’s ambivalence in balancing the costs and benefits of possible changes. As such, evoking an individual to find solutions on their own is encouraged under this approach. M.I., originally a micro-counseling technique in clinical settings (32), has successfully facilitated lifestyle changes, such as physical activity (33), treatment adherence (34), teen pregnancy prevention, and fitness behavior (35).

Motivational interventions do not have to be face-to-face. In fact, written motivational messaging is common and can take the format of written guidelines (36), web-based interventions (37), text messages (38), and leaflets (39). Encouraging individuals to visit nature by using written motivational messages aligns with the self-determination theory’s need for autonomy and connectedness. If written motivational messaging is integrated with realistic expectations, and if the ambivalence is addressed in M.I., the approach should possess motivational properties.

Nature prescription becomes a trend for mental health problems given that traditional psychiatric and psychological approach like cognitive-behavioral therapy have their weakness particularly on the maintenance of the effect (40–42). While simple nature exposure, such as sitting or walking outdoors, have been shown to reduce stress and anxiety compared to control settings, the use of motivational technique to suggest more nature exposure has not been examined for enhancing the mental health benefits. We targeted on essential elements of M.I. which was shown to be an evidence based approach to increase treatment adherence. We conducted a between-subjects study in which we randomly assigned young adults recruited from universities in Taipei, Taiwan to an experimental group that received messaging about nature-related stress relief measures plus MI, compared with three control groups.

## Materials and methods

### Our research questions are

Can reading stress-relief (nature) messages together with motivational enhancement questions increase participant motivation for nature exposure and result in benefits to their wellbeing? Three other control conditions for comparison read: (1) Non-stress-relief message (2) Stress-relief (non-nature) message, and (3) Stress-relief (nature) message without answering the motivational enhancement questions. In view of the sudden outbreak of the pandemic, we grasped the opportunity to answer the following questions as well:

During different stages of the COVID-19 pandemic (pre, 1 week, and 1 month), how much variance in connectedness to nature is explained by the indicators of motivation for nature exposure?

During the COVID-19 pandemic, how much variance in perceived stress can be explained by connectedness to nature?

### Define life stressor to the participants

This research originally targeted stress experienced by university students regarding studying. Its pre–test covered the period from 2nd May 2021 to 15th May 2021. Unfortunately, an epidemic escalation occurred on 15th May 2021 in Taipei, in the northern part of Taiwan, with classes and exam suspended. Our study’s 1 week post-test took place after 16th May, which followed the lockdown of the city. The study’s timeline coincided with the development of the pandemic in Taiwan. After outbreak of the pandemic, we asked participants to recall three impressive images from their daily lives. We examined these items and categorized the images. Of the 540 total images the participants recalled 1 week and 1 month post experiment, 133 images were related to the pandemic, while 54 were related to study stress, resulting in a significant difference [*χ^2^*(1) = 34.04, *p* < 0.01]. We concluded the pandemic was the main concern to participants during our present study and, appropriately, have the pandemic as our research focus and primary discussion topic.

### Participants

University students aged 21 to 35 years old were recruited from an online social media platform. We selected this age range because the term “young people” is used interchangeably for ages 15–24 and can be extended in some cases to 30 or 40 years old (43). Figure one illustrates the flow and drop out of the study. The final sample size was 90 (mean age = 23.41 years, SD = 2.40). For gender identity, 63.3% were female (mean age = 23.28 years, SD = 2.48) and 36.7% were male (mean age = 23.64 years, SD = 2.28). Most participants came from National Taiwan University (45.6%), National Taiwan Technology University (13.2%), National Taiwan Normal University (5.5%), and Yang Ming University (5.5%), and the others were spread across 20 universities. Regarding program of study, 73.3% were studying in a Bachelor’s program and 26.7% were in a Master’s program. The four experimental groups were not significantly different in age *F*(3, 86) = 1.13, *p* > 0.05, gender *χ^2^*(3, 90) = 3.79, *p* > 0.05, or affiliated academic programs (i.e., Bachelor vs. Master) *χ^2^*(3, 90) = 0.77, *p* > 0.05.

### Program content

**FIGURE 1:**
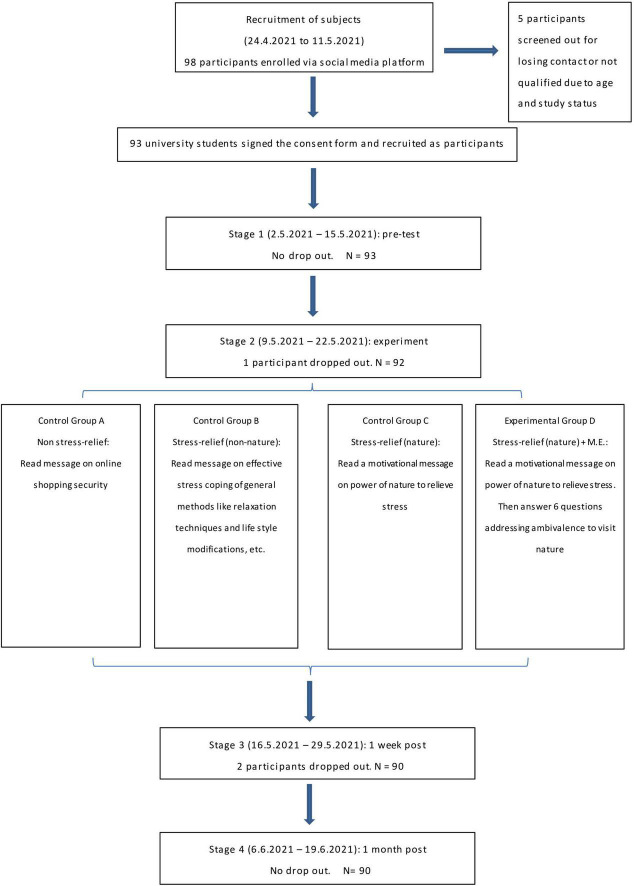
The flow of the study and its drop out at different stages.

After being recruited, the participants were given a written explanation of the stages and content of the study. See Figure 1 for the flow of the study and drop out at different stages. Participants had to sign a consent form before the study started. The participants were asked to access online materials *via* QR code that was sent to their social media account during the experimental sessions (fixed on a particular weekday). For stages 1, 3, and 4, they were given a questionnaire to complete. For stage 2, the participants read a passage and answered questions. Throughout the four stages of the study, the participants had to return the questionnaires within 12 h. Failure to do so would lead to disqualification of the participant. The participants could terminate participation in the study at any time. Upon completion of the study and verification of identity online, $400 in Taiwanese dollar payments were deposited in participant bank accounts. Such minimal monetary return, equivalent to one movie ticket in Taiwan’s cinema, should be regarded as imposing very low influence on the motivation of the participants and hence confounding the study very minimally. All the data collected was kept confidential and was used only for analysis in this study. The study had been approved by the Research Ethics Committee of National Taiwan University.

For all measurements, participants were to answer the questions based on what they had experienced during the past week, except for the Perceived Stress Scale which concerned conditions the past month. We gave the participants a soft reminder 1 h before questionnaire submission deadlines. After receiving a questionnaire, we immediately examined the answers to check for signs of a response set. If a response set was found, we contacted the participants within an hour to confirm the validity of their responses and to check whether they wanted to revise the answers. For the entire study, there were nine suspected response set replies, among which four were subsequently revised by its participant.

As for the written messages of the four experimental conditions, we drafted them and then piloted them on five young adults before the study to ensure readability and to collect feedback on whether the messages motivated them to seek nature exposure. Feedback from the pilot was considered, and we incorporated the final messages into a 1500-word Chinese article. During stage 2 of the study, the participants received a specific message depending upon which group they belonged to. They answered five questions assessing their comprehension of the message, and those that answered two or more questions incorrectly would be disqualified (and their data would not be further analyzed). All participants passed the comprehension test.

The content of the messages was as follows:

Control group A (*n* = 23): Non-stress relief.

The reading is related to online shopping security with information consolidated from the web.

Control group B (*n* = 21): Stress relief (non-nature).

The reading addresses the stress of students empathetically at the beginning and then summarizes generally effective stress coping techniques, which include relaxation, connection with a support network, positive thinking, and lifestyle modification. The content was written by one of the researchers, who is a clinical psychologist, with reference to Alborzkouh et al. (44) review of the effectiveness of stress management skills.

Control group C (*n* = 23): Stress relief (nature).

The reading is about the power of nature in relieving stress. It begins expressing empathy for the stress of students and then summarizes research findings of how a brief nature experience can induce a physiological stress reduction effect (18). It describes how autonomous, restorative, and inspiring it can be to simply expose oneself to nature. The information presented resembles self-determination theory’s autonomy, self-efficacy, and connectedness while also addressing optimistic yet realistic outcome expectancy.

Experimental Group D (*n* = 23): Stress relief (nature) + M.E.

They received the same reading material as group C. However, after finishing the comprehension test, the participants had to fill in six open-ended motivational enhancement (M.E.) questions on how to resolve scenarios that hindered visits to nature. Making reference to an integrated systematic review on barriers to nature exposure (45), we developed questions addressing traveling time, cost, difficulty finding proper nature sites, weather and insect interference, and boredom while in nature. Although the inspiring questions were presented as a case study, they were really for the participants to resolve their possible ambivalence toward nature exposure. Use of a case study approach was to minimize side-taking of conflicts and the subsequent escalation of resistance (31) arising from first person perspective. Hopefully the above measures generated an inviting atmosphere rather than pressure on the participants to adopt new ways of thinking. Also, we intentionally guided participants’ preference toward nature by asking them to recall a positive memory involving nature.

### Measurements

#### Motivation for nature visit

Because there was no available established measurement of motivation concerning nature exposure, we had to develop our own assessment tool for this domain. We attempted to measure motivation in a multi-construct manner according to three fields of motivation, namely cognitive, emotional, and behavioral (46), with a single question for each indicator. While single-item assessments have been criticized for their shortcomings of higher susceptibility to random measurement error (47), there is evidence it has equal predictive utility as compared to multi-item measures [e.g., (48–50)]. For the emotional aspect, we used “I intend to,” since Fishman et al. (51) reported this expression may be optimal for predicting specific behavior. For questions on belief and intention, we asked participants to answer on a seven-point Likert scale.

Cognitive: Belief that exposure to nature can relieve stress.

Question: “Exposure to nature can relieve stress.” To what degree do you agree with this statement?

Emotional: Intend to expose oneself to nature for stress relief.

Question: “To what degree do you intend to expose yourself to nature for stress relief?”

Behavioral: Referencing Hunter et al. (18) study, we set the time standard to 20 min. We were aware the answers to this question could be a mixture of visiting nature alone and visiting nature with family and friends. For visiting nature with family and friends, it could be for a social purpose apart from a stress relief purpose. Therefore, this question was further broken down into two:

Frequency of exposure to nature for at least 20 min in last week on one’s own.

Question: “In the last week, how many times have you exposed yourself to nature alone for at least 20 min?”

Frequency of exposure to nature for at least 20 min in last week with family and friends.

Question: “In the last week, how many times you have exposed yourself to nature with family and friends for at least 20 min?”

#### Motivation for virtual nature visit

Because the pandemic may have reduced visits to nature while online activities likely increased for young adults, we added three questions regarding virtual nature visits for stages 3 and 4. The questions were parallel to similar questions concerning nature exposure.

Cognitive: Belief exposure to virtual nature can relieve stress.

Question: “Exposure to virtual nature can relieve stress.” To what degree do you agree with this statement?

Intend to expose oneself to virtual nature for stress relief.

Question: “To what degree do you intend to expose yourself to nature for stress relief?”

Frequency of exposure to virtual nature for at least 20 min the past week.

Question: “In the past week, how many times have you exposed yourself to virtual nature for at least 20 min?”

Referring to Hunter et al. (18), we originally defined nature experience as “anywhere outside that, in the opinion of the participant, included a sufficiency of natural elements to feel like a nature interaction.” We surveyed ten young Taiwanese adults, and eight of them indicated this definition of nature experience was too broad or too vague for them. This perhaps may be due to Chinese preference for concrete stimuli rather than abstract stimuli (52). Having taken the pilot participants’ advice to offer more guidance for the definition, in the present experiment we clarified nature experience as “outdoor space that grants you a sense of nature, including mountain, water, sky, plants or animals, the songs of the birds or sounds of insects, and even a grassland ….. The ellipsis is to render it a non-inclusive description for the participants to elaborate or imagine for themselves. For the virtual nature, “virtual” refers to media information or videos, and “nature” assumes the same definition as aforementioned.

To avoid polarization impact by response outliers, we studied the descriptive statistics and recategorized the answers into four categories, with frequencies of zero to two being the same as the actual frequency, and frequencies of three or above being classified as three. This categorization applied to all questions with frequency counts.

#### Recall of three impressive images in the last week

As the pandemic and resulting social distancing likely hindered the participants physically connecting with nature, we innovatively constructed an item at 1 week and 1 month post-test to hopefully capture how often the participants had tended to nature and its positive images. The participants recalled three images from their daily lives and stated whether the images brought them positive, negative, or mixed/uncertain feelings. The instructions for this question were as follows:

“Our eyes are comparable to a camera lens while our heads are comparable to hard disks of our memory. Whether these images are from indoors or outdoors, they can elicit feelings. Please recall images from your daily living that brought you feelings in the past week. Write three of these images, describe them in detail, and then determine whether they brought you positive, negative, or mixed/uncertain feelings.”

We randomly selected 50 images recalled by the participants and had them scored independently by two scorers who had a psychology background and were pursuing a Master’s degree or higher in the Forestry School. The scorers decided whether the images related to nature or not by referring to Hunter et al. (18) definition of a “nature-experience.” Of the 50 images, 48 were classified in the same category. The inter-rater reliability was regarded as high, which made us feel comfortable with the scoring criteria. Once the two inconsistent ratings were resolved, one of the scorers completed the remaining categorizations.

Six categories were yielded from the three (positive/negative/mixed) × two (nature/non-nature) dimensions. Since feelings are subjective, we aimed not to edit the classifications of the participants unless there was an absolute doubt of the classification. In total, seven positive image statements and four negative image statements were found to be problematic. Examples of the problematic positive statements are: “Empty high-speed rail makes seating spacious and comfortable. Unfortunately, I still have to return home” and “The roses that I have planted for half a year pecked off by birds thrice before it blooms. It’s beautiful but I feel sad for it.” Examples of the problematic negative statements are: “The weather is cloudy, but it makes me feel cool!” and “There was a prolonged drought. Watching the rain infiltrates the ground, I can feel the prosperity.” These items carried mixed feelings and, therefore, were reclassified as mixed/uncertain by the researchers. The categories entered into statistical analysis were: (1) number of recalls of positive nature stimuli (i.e., positive nature), (2) number of recalls of positive stimuli (i.e., positive nature + positive_non-nature) (3) number of recalls of nature stimuli (images about nature regardless of it being positive, negative, or mixed/uncertain one).

While research involving recall like autobiographical memory [e.g., (53, 54)] and narrative study [e.g., (55, 56)] are not uncommon to elicit one’s memory of past experience, the use of recall as a measurement should be very careful due to its validity. The concerns are individuals’ memory of past mood, emotions, cognitions, and behaviors seem to do more with the reconstructions combing opinions, behaviors and thoughts of an individual than pure retrieval (57–61). In this study, the measurement capturing memory of daily life was added in an ad-doc manner as the pandemics may affect human’s reaching out to the nature and hence hinder the experimental effect captured by planned measurements. We therefore thought it was worth to pilot the memory measurement in such a special situation. We followed the Autobiographical Memory Test [AMT; (62)] to suggest the participants to state as many details as they could in relation to an impressive event. However what being different from AMT was that we did not confine the participants’ recall by cued words. In such a way we can tap into what really catch the attention of the participants in daily life. Also, requiring the participants to make the recall for last week is of advantageous given that research had shown under short delay (1 week) the participants were more capable to recall details of a memory than long delay (1 month) (63). Our target population on young adult was also favorable for eliciting distinctive memory after participation in the experiment given that young adult, when compared with older adults, was found to detect and remember more changes (64). Finally but also hopefully, we assume that the problem to do with memory bias should have been even out under random subject assignment to the experimental groups. See Appendix for “Measurements on motivation for nature visits” and “Recall of three impressive images in the last week”.

#### Other measurements

##### Wellbeing

We wanted to explore whether the wellbeing of the participants was impacted under the different experimental conditions.

Perceived Stress Scale-14 items (PSS-14) (1 week pre and 1 month post).

The Perceived Stress Scale (PSS) (65), a commonly used psychological instrument indicating the perception of stress, is rated on a five-point Likert scale. A recent systematic review of more than 40 experimental studies found measures of perceived stress grant convincing evidence of the relationship between exposure to nature and reduced stress levels (12). PSS’s Cronbach’s alpha was 0.85 in a study of Taiwanese adults (66) and 0.77 in a study of stress, active coping, and problem behaviors among Chinese adolescents (67), indicating its good reliability. During the COVID-19 pandemic, PSS scores among healthcare workers in China were higher than a cut-off value (68), revealing the scale is sensitive to stress in the Chinese population related to the pandemic. The present study employed the validated Chinese version of PSS by Chu and Kao (66).

Depression Anxiety Stress Scale-21 items (DASS-21) (1 week pre, 1 week post, and 1 month post).

To measure participant response to stress, we employed the Depression Anxiety Stress Scale-21 (DASS-21) (69) to supplement PSS-14. The questions are rated on a four-point Likert scale. Good psychometric properties of the scale have been demonstrated in studies (70, 71). The three factor structure of the scale has been validated in non-clinical (72) apart from clinical samples (73). DASS-21’s convergent validity coefficient was 0.87 in a young adult population experiencing psychological distress (74). Chinese DASS-21 was found to be effective in differentiating between depression, anxiety, and stress, and it is suitable for regular assessment and treatment evaluation (75). The Chinese-translated scale employed in this study is from Moussa et al. (76).

Flourishing scale (FS) (1 week pre, 1 week post, and 1 month post).

Studies on nature and mental health should focus more on positive health, such as happiness, purpose, and flourishing, rather than just the absence of negative mental health outcomes (17). Thus, flourishing was measured in the present study. The flourishing scale (FS), formerly known as the psychological wellbeing scale (PWBS), consists of eight items. They are rated on a six-point Likert scale for measuring respondents’ self-perceived successes in relationships, self-esteem, purpose, and optimism (77). The scale has a high internal consistency (α = 0.87), high 1-month test–retest reliability (*r* = 0.71), a robust single-factor structure in EFA, and good criterion-related validity with basic life satisfaction (*r* = 0.78) and psychological wellbeing (*r* = 0.73) (77). During the pandemic, the fear of COVID-19 correlated significantly with FS at −0.16 for a student population (78). The present study employed the translated Chinese version of PWB, which was found to be above 0.90 in Cronbach’s alpha and of adequate fit indices for a single-factor model (79).

Connectedness to Nature Scale (CNS) (1 week pre, 1 week post, and 1 month post).

The Connectedness to Nature Scale (CNS) (80), a popular instrument for tapping this construct, was employed as an indicator of success for our motivational strategy to connect young adults with nature. The scale assesses the “experiential sense of oneness with the natural world” [(80) p. 504], or the sense of whether people feel part of their surrounding natural world. As a 14-items scale that is rated on five-point Likert scale, CNS has only one factor and possesses high internal consistency (α = 0.84) and test–retest reliability (*r* = 0.79) (80). CNS is inversely correlated with perceived stress (*r* = -0.16, *p* = 0.01), anxiety (*r* = -0.11, *p* = 0.04), and depression (*r* = -0.15, *p* = 0.04) (81). It correlates significantly with FS at 0.31 (82). The present study employed Li and Cao (83) translated version of CNS.

##### Control variable

Multidimensional Scale of Perceived Social Support Scale (MSPSS) (1 week pre, 1 week post, and 1 month post).

Since a low perceived social support could have a negative impact on psychological symptoms during the COVID-19 pandemic (8), we used the 12-item Multidimensional Scale of Perceived Social Support rated on a seven-point Likert scale [MSPSS; (84)] to measure as a control variable participants’ perceived social support. In a study of college students, MSPSS correlated significantly with the DASS subscales of depression (−0.34), anxiety (−0.14), and stress (−0.22) (85). While there are three sources of support specified in the scale, namely family, friends, and significant others, we used the combined total score as an index for general social support; higher scores indicate more perceived social support. We employed the validated translated scale of Chou (86), which has good internal consistency (0.89) and correlated negatively with depression and anxiety in a sample of Chinese adolescents.

## Methods

We followed the rule of thumb of 20 + 5k (87), with k being the number of predictors, to govern our selection of variables to be put in our multiple regression model. We performed *a priori* sample size calculation [G*Power; (88)] with an estimation of main treatment effect of 0.50, assuming a significance level (alpha) of 0.05, and a statistical power (1-beta) of 80%. This calculation indicated 48 participants would be required for ANOVA. The study’s actual number of participants, 90, was regarded as sufficient for performing statistical analyses of repeated measurements, ANOVA and hierarchical regression. The data were analyzed using SPSS statistics 27. Correlations for all the measures, including the self-constructed items and the employed instruments, had been performed.

We conducted three time (pre/1 week/1 month) × four groups repeated-measures ANOVA on “Belief in exposure to nature can relieve stress,” “Intend to expose oneself to nature for stress-relief,” “Frequency of exposure to nature for at least 20 min in last week on one’s own,” and “Frequency of exposure to nature for at least 20 min in last week with family and friends” to explore the groups’ effects on motivation to be exposed to the nature. We adjusted the degrees of freedom to Greenhouse-Geisser (when Epsilon of Greenhouse-Geisser was <0.75) or Huynh-Feldt (when Epsilon of Greenhouse-Geisser was >0.75) when the test of sphericity was significant (89). For belief, intention, and frequency of exposure to the virtual nature, as well as for recalls of “Positive Nature,” “Positive,” and “Nature” images, only 1 week and 1 month post data were available because we did not initially plan to ask these questions. One way between-subjects ANOVA was conducted instead on these items.

For the wellbeing impacts of the different experimental conditions, one way between-subjects ANOVA were conducted for PSS, which only had two measurements (i.e., pre and 1 month post), while three Time (pre/1 week/1 month) × four Groups repeated-measures ANOVA on DASS Total, DASS_Stress, DASS_Anxiety, DASS_Depress, and Flourishing, and three Time (pre/1 week/1 month) × four Groups repeated-measures ANOVA on CNS had been performed.

To approach the research question of whether connectedness to nature can be explained by belief, intention, and behavior of exposure to nature, hierarchical linear regression analyses were conducted across the different time measurements. For behavior exposure, we put both the frequency of exposure and the recall of positive images of nature into the model at 1 week and 1 month post because they had yielded valuable findings in the prior ANOVA analyses.

Finally, a Hierarchical Multiple Regression was performed to examine the role of Connectedness to Nature in explaining PSS with gender, age, group membership, pre-score of PSS, and pre-score of the MSPSS being controlled for.

## Results

As noted in Table 1, the mean response scale values were calculated for all measures and all times. The reliability of each scale was acceptable and some attained an excellent level as indicated by Cronbach’s α.

**TABLE 1:** Mean, standard deviation and reliability of the scales employed in the study (*N* = 90).

	Time	Reliability	Control group A (*n* = 23)		Control group B (*n* = 21)		Control group C (*n* = 23)		Experimental group D (*n* = 23)	
						
		α	*M*	SD	*M*	SD	*M*	SD	*M*	SD
**Inventory**										
Perceived stress scale (14 items)	1w pre	0.84	31.09	7.23	28.33	7.68	29.96	7.40	31.83	7.26
	1 m post	0.74	33.22	7.66	30.90	11.40	32.35	8.26	24.52	12.79
Depression, anxiety and stress scale (21 items)	1wpre	0.89	19.22	9.50	16.38	10.32	16.30	9.99	15.13	9.03
	1w post	0.90	18.65	10.50	15.19	8.95	14.91	9.37	14.64	10.51
	1 m post	0.92	17.52	11.14	16.43	10.60	16.96	9.75	15.91	12.44
Flourishing scale (8 items)	1w pre	0.86	37.91	7.79	37.48	8.71	37.22	6.41	35.96	7.23
	1w post	0.85	37.35	6.12	38.10	8.43	37.78	5.80	36.57	7.26
	1 m post	0.89	35.52	8.90	36.71	8.98	36.91	6.35	37.39	7.41
Connectedness to nature scale (14 items)	1w pre	0.89	36.30	8.82	33.57	8.29	32.04	7.35	32.61	9.89
	1w post	0.86	36.74	8.70	34.90	7.65	35.40	7.07	35.04	10.64
	1m post	0.88	36.35	11.15	35.14	6.97	36.04	6.48	35.91	10.62
Multi-dimensional scale of perceived social support (12 items)	1w pre	0.88	59.30	9.82	60.71	11.52	57.74	12.81	60.61	11.33
	1w post	0.91	60.91	10.73	62.29	12.03	60.09	10.02	62.22	11.43
	1m post	0.93	61.43	13.20	60.95	11.76	59.04	11.26	62.57	12.46

### General findings

Table 2 contains correlations for all the measures in the present study. The correlations of the same battery of tests across three measurements were each above or approaching 0.80 (<0.01), except for PSS pre and post, which were only 0.50 (<0.01). Table 3 contains correlations across three measurements for the self-constructed items regarding belief and intention related to nature or virtual nature. Interestingly, we could nearly note the emergence of distinct correlations for the categories of “Nature” and “Virtual nature,” with items belonging to the same category significantly correlating. The two categories did not correlate with each other except at 1 month post when “Intend to expose oneself to virtual nature”’ was significantly correlated with “Belief in exposure to nature can relieve stress” 0.29 (*p* < 0.01) and “Intend to expose oneself to nature for stress-relief” 0.30 (*p* < 0.01). The major correlations for items belonging to the same category support the validity of these constructed motivational questions.

**TABLE 2:** Correlations of inventories employed in the present study (*N* = 90).

	PSS_ 1w pre	PSS_1m post	DASS_1w pre	DASS_1w post	DASS_1m post	FS_1w pre	FS_1w post	FS_1m post	CNS_1w pre	CNS_1w post	CNS _1m post	MSPSS _1w pre	MSPSS _1w post	MSPSS_1m post
PSS_ 1w pre	1													
PSS_1m post	0.508**	1												
DASS_ 1w pre	0.551**	0.466**	1											
DASS_1w post	0.538**	0.515**	0.758**	1										
DASS_ 1m post	0.482**	0.568**	0.678**	0.802**	1									
FS_1w pre	−0.457**	−0.420**	−0.443**	−0.524**	−0.542**	1								
FS_1w post	−0.478**	−0.387**	−0.425**	−0.586**	−0.605**	0.776**	1							
FS_1m post	−0.396**	−0.486**	−0.471**	−0.580**	−0.676**	0.747**	0.817**	1						
CNS__1w pre	−0.130	−0.178	−0.136	−0.205	−0.153	0.354**	0.294**	0.233*	1					
CNS_1w post	−0.193	−0.214*	−0.192	−0.246*	−0.190	0.355**	0.404**	0.310**	0.838**	1				
CNS_ 1m post	−0.167	−0.293**	−0.253*	−0.327**	−0.305**	0.330**	0.359**	0.390**	0.752**	0.868**	1			
MSPSS _ 1w pre	−0.242*	−0.182	−0.227*	−0.266*	−0.317**	0.589**	0.564**	0.524**	0.194	0.220*	0.183	1		
MSPSS _ 1w post	−0.276**	−0.214*	−0.192	−0.232*	−0.328**	0.551**	0.652**	0.528**	0.136	0.216*	0.188	0.819**	1	
MSPSS _ 1mpost	−0.262*	−0.283**	−0.212*	−0.273**	−0.443**	0.582**	0.656**	0.653**	0.098	0.205	0.227*	0.791**	0.884**	1

**Correlation significant at the 0.01 level (2-tailed).

*Correlation significant at the 0.05 level (2-tailed).

1w: 1 week; 1m: 1 month.

PSS, Perceived Stress Scale-14 items; DASS, Depression Anxiety Stress Scale-21 items; FS, Flourishing Scale; CNS, Connectedness to Nature Scale; MSPSS, Multidimensional Scale of Perceived Social Support Scale.

**TABLE 3:** Correlations of self-constructed items related to motivation (*N* = 90).

	Belief in nature can relieve stress_1w pre	Intend to expose oneself to nature for stress-relief _1w pre	Belief in nature can relieve stress _1w post	Belief in virtual nature can relieve stress_1w post	Intend to expose oneself to nature for stress-relief _1w post	Intend to expose oneself to virtual nature for stress-relief_1w post	Belief in nature can relieve stress _1m post	Belief in virtual nature can relieve stress_1m post	Intend to expose oneself to nature for stress-relief_1m post	Intend to expose oneself to virtual nature for stress-relief _1m post
Belief in nature can relieve stress_1w pre	1									
Intend to expose oneself to nature for stress-relief _1w pre	0.560**	1								
Belief in nature can relieve stress _1w post	0.158	0.236*	1							
Belief in virtual nature can relieve stress_1w post	0.106	0.127	−0.107	1						
Intend to expose oneself to nature for stress-relief _1w post	0.290**	0.422**	0.541**	−0.066	1					
Intend to expose oneself to virtual nature for stress-relief_1w post	0.098	0.132	−0.077	0.434**	0.054	1				
Belief in nature can relieve stress _1m post	0.256*	0.250*	0.623**	0.008	0.531**	0.132	1			
Belief in virtual nature can relieve stress_1m post	0.165	0.088	−0.195	0.619**	−0.150	0.233*	0.109	1		
Intend to expose oneself to nature for stress-relief_1mpost	0.285**	0.344**	0.237*	0.034	0.496**	0.182	0.579**	0.108	1	
Intend to expose oneself to virtual nature for stress-relief _1m post	0.154	0.163	0.143	0.271**	0.075	0.587**	0.291**	0.488**	0.296**	1

**Correlation is significant at the 0.01 level (2-tailed).

*Correlation is significant at the 0.05 level (2-tailed).

1w: 1 week; 1m: 1 month.

Table 4 displays correlations for the self-constructed items concerning the frequency of exposure to nature or virtual nature for at least 20 min in the past week. The clustered pattern of correlation is less obvious but still grossly distinguishes between frequency of nature exposure on one’s own vs. nature exposure with family and friends vs. access *via* virtual means, supporting the validity of the questions in tapping into different behaviors. Interestingly, exposure to virtual nature 1 month post significantly correlated with 1 week and 1 month frequency of nature exposure on one’s own and with family and friends. This may be because virtual nature visits replaced actual nature visits during the pandemic.

**TABLE 4:** Correlations of self-constructed items on frequency of exposure to nature or virtual nature for at least 20 min in last week (*N* = 90).

	Exposure to nature with family/friends_1w pre	Exposure to nature on one own_1w pre	Exposure to nature with family/friends_1w post	Exposure to nature on one own_1w post	Exposure to virtual nature_1w post	Exposure to nature with family/friends_1m post	Exposure to nature on one own_1m post	Exposure to virtual nature _1m post
Exposure to nature with family/friends_1 w pre	1							
Exposure to nature on one own_1 w pre	0.158	1						
Exposure to nature with family/friends _1w post	0.288**	0.187	1					
Exposure to nature on one own_1w post	0.131	0.232*	0.162	1				
Exposure to virtual nature_1w post	0.304**	0.077	0.411**	0.100	1			
Exposure to nature with family/friends _1m post	0.059	0.136	0.125	0.113	0.162	1		
Exposure to nature on one own_1m post	0.063	0.362**	0.053	0.024	0.016	0.053	1	
Exposure to “virtual” nature_1m post	0.251*	0.347**	0.340**	0.347**	0.440**	0.070	0.198	1

**Correlation is significant at the 0.01 level (2-tailed).

*Correlation is significant at the 0.05 level (2-tailed).

1w: 1 week; 1m: 1 month.

### Research question 1: Group effect on motivation indicators and wellbeing

#### Group effect on motivation indicators related to exposure to nature

**FIGURE 2:**
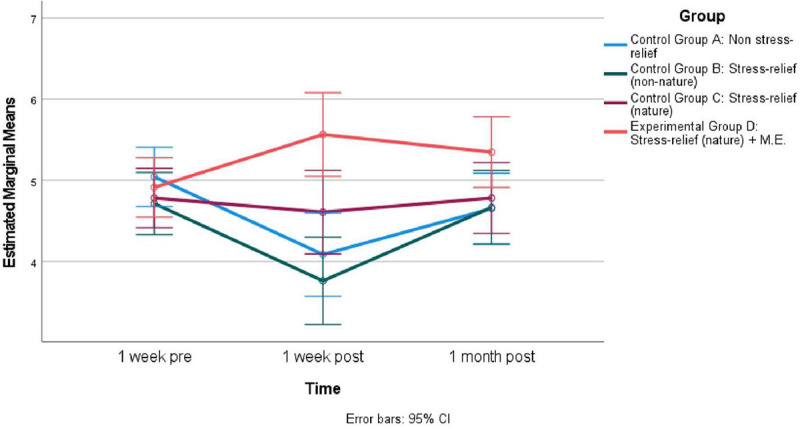
Estimated marginal means of “Belief in nature can relieve stress” across different time for four groups (*N* = 90).

The repeated measures ANOVA on “Belief in nature can relieve stress” yielded a significant effect for time *F*(1.89, 162.87) = 5.15, *p* < 0.01, Eta^2^ = 0.06 and the interaction between time and group *F*(5.68, 162.87) = 4.75, *p* < 0.01, Eta^2^ = 0.14. This reflects that the changes in “Belief in nature can relieve stress” over time among the different groups differed. Results of a pairwise comparison suggest the four groups were similar in “Belief in nature can relieve stress” at the beginning of the study. However, 1 week after the experiment, “Stress-relief (nature) + M.E. “Group were higher in “Belief in nature can relieve stress” than the “Stress-relief (non-nature)” Group (MD = 1.80, SE = 0.38, *p* < 0.01) and the “Non-stress-relief” Group (MD = 1.48, SE = 0.37, *p* < 0.01). One month after the experiment, there were not any significant differences among the four groups. See Figure 2 for Estimated Marginal Means of “Belief in nature can relieve stress” for the four groups 1 week pre-experiment, 1 week post, and 1 month post.

**FIGURE 3:**
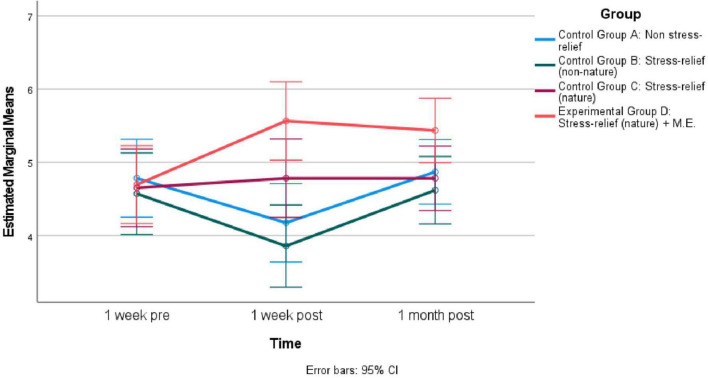
Estimated marginal means of “Intend to expose oneself to nature for stress-relief” across different time for four groups (*N* = 90).

The repeated measures ANOVA on “Intend to expose oneself to nature for stress-relief” yielded a significant effect for time *F*(2, 172) = 3.14, *p* < 0.05, Eta^2^ = 0.04 and the interaction between time and group *F*(6, 172) = 3.78, *p* < 0.01, Eta^2^ = 0.12. This reflects the changes in “Intend to expose oneself to nature for stress-relief” over time among the different groups differed. Result of a pairwise comparison suggest the four groups were similar in “Intend to expose oneself to nature for stress-relief” at the beginning of the study. However, 1 week after the experiment, the “Stress-relief (nature) + M.E. “Group were higher in “Intend to expose oneself to nature for stress-relief” than the “Stress-relief (non-nature)” Group (MD = 1.71, SE = 0.39, *p* < 0.01) and the “Non-stress-relief” Group (MD = 1.39, SE.38, *p* < 0.01). One month after the experiment, there were not any significant differences among the four groups. See Figure 3 for Estimated Marginal Means of “Intend to expose oneself to nature for stress-relief” for the four groups across 1 week pre-experiment, 1 week post, and 1 month post.

For “Frequency of exposure to nature for at least 20 min in the past week on one’s own,” there was not a significant effect for time *F*(1.87, 161.03) = 1.87, *p* > 0.05, Eta2 = 0.02 or time × group *F*(5.62, 161.03) = 0.88, *p* > 0.05. For “Frequency of exposure of nature for at least 20 min in the past week with family and friends,” there was a significant effect for time *F*(2, 172) = 1.39, *p* > 0.05, Eta2 = 0.12 but not for time × group *F*(6, 172) = 1.40, *p* > 0.05, Eta2 = 0.05. In general, participants experienced a decrease in their “Frequency of exposure of nature for at least 20 min in last week with family and friends” from pre experiment to 1 month post experiment, regardless of their group membership. This is likely explained by the pandemic hindering social activities in nature.

The above findings reflect that young adults only had more favorable attitudes toward exposure to nature in 1 week, measured by belief and intention, when compared with the two control groups, namely “Non-stress-relief” and “Stress-relief (non-nature),” when prompted to answer motivational enhancement questions after reading their motivational message. Group membership failed to make a difference on the actual frequency of exposure to nature, regardless of whether exposure was on one’s own or with family and friends.

#### Group effect on motivation to be exposed to virtual nature

Table 5 shows the one way between-subjects ANOVA for belief, intention and frequency of exposure to the virtual nature.

**TABLE 5:** One way ANOVA on PSS, virtual items and recall items, comparing experimental group with three control groups (*N* = 90).

Domain		ANOVA			*Post hoc* test by Turkey HSD comparing “Stress-relief (nature) + M.E.”^a^ with				
		
	Pre/post	df	Between group Mean^2^	F	Control group^b^	MD (a minus b)	Standard error	Upper bound	Lower bound
PSS	1w pre	3, 86	50.52	0.93	A	0.74	2.18	−4.97	6.45
					B	3.49	2.23	−2.35	9.33
					C	1.87	2.18	−3.84	7.58
	1m post	3, 86	355.81	3.41*	A	−8.70*	3.01	−0.*16*.59	7.03
					B	−6.38	3.09	−14.47	1.7
					C	−7.83	3.01	−15.72	0.07
Belief in virtual nature can relieve stress	1w post	3, 86	7.57	6.28**	A	−1.17**	0.32	−2.02	−0.33
					B	−1.27**	0.33	−2.14	−0.41
					C	−0.74	0.32	−1.59	0.11
	1m post	3, 86	8.67	6.66**	A	−1.22**	0.34	−2.1	−0.34
					B	−1.37**	0.34	−2.27	−0.47
					C	−0.65	0.34	−1.53	0.23
Intend to expose oneself to virtual nature for stress-relief	1w post	3, 86	6.99	3.87*	A	−1.09*	0.4	−2.13	−0.05
					B	−1.26*	0.41	−2.32	−0.19
					C	−0.83	0.4	−1.86	0.21
	1m post	3, 86	4.15	1.84	A	−0.87	0.44	−2.03	0.29
					B	−0.94*	0.45	−2.13	0.25
					C	−0.52	0.44	−1.68	0.64
Frequency of exposure to virtual nature for at least 20 min in last week	1w post	3, 86	0.94	0.92	A	−0.09	0.3	−0.87	0.69
					B	−0.15	0.3	−0.95	0.64
					C	−0.04	0.3	−0.82	0.74
	1m post	3, 86	1.27	1.21	A	−0.27	0.3	−1.05	0.54
					B	−0.53	0.31	−1.34	0.28
					C	−0.04	0.3	−0.84	0.75
Number of recall on positive image of nature	1 week post	3, 86	2.74	3.64*	A	0.78*	0.26	0.11	1.45
					B	0.3	0.26	−0.39	0.98
					C	0.61	0.26	−0.06	1.28
	1 month post	3, 86	2.18	3.56*	A	0.65*	0.23	0.05	1.26
					B	0.45	0.24	−0.17	1.07
					C	0.65*	0.23	0.05	1.26
Number of recall on positive image	1 week post	3, 86	1.82	2.34	A	0.65	0.26	−0.03	1.33
					B	0.21	0.27	−0.49	0.91
					C	0.43	0.26	−0.25	1.12
	1 month post	3, 86	1.7	3.10*	A	0.35	0.22	−0.22	0.92
					B	0.22	0.22	−0.36	0.81
					C	0.65*	0.22	0.08	1.22
Number of recall on nature image	1 week post	3, 86	1.29	1.22	A	0.49	0.3	−0.32	1.27
					B	0.1	0.31	−0.71	0.92
					C	0.43	0.3	−0.36	1.23
	1 month post	3, 86	1.01	1.21	A	0.22	0.27	−0.49	0.92
					B	−0.08	0.28	−0.8	0.64
					C	0.39	0.27	−0.31	1.1

Control Group A: Non-stress-relief. Control Group B: Stress-relief (non-nature).

Control Group C: Stress-relief (nature).

*p < 0.05; **p < 0.01.

^a^Stress-relief (nature) + M.E. group.

^b^The corresponding control group.

Regarding “Belief in virtual nature can relieve stress,” there were significant differences between the four groups both 1 week post and 1 month post. As indicated by the *post hoc* comparisons made using the Turkey HSD test, “Stress-relief (nature) + M.E.” Group (1 week post: *M* = 2.87, SD = 1.42, 1 month post: *M* = 2.87, SD = 1.39) was significantly lower than “Non-stress-relief” Group (1 week post: *M* = 4.04, SD = 1.07; 1 month post: *M* = 4.09, SD = 1.28) and “Stress-relief (non-nature)” Group (1 week post: *M* = 4.14, SD = 0.06; 1 month post: *M* = 4.24, SD = 0.70). For “Intend to expose oneself to virtual nature for stress-relief,” significant differences were found among the four groups at 1 week post. A pairwise *post hoc* test revealed “Stress-relief (nature) + M.E. “Group (*M* = 2.70, SD = 1.66) was significantly lower than “Non-stress-relief” Group (*M* = 3.79, SD = 1.09) and “Stress-relief (non-nature)” Group (*M* = 3.96, SD = 0.97). However, the significant differences among the groups no longer existed at 1 month post. For “Frequency of exposure to virtual nature for at least 20 min in last week at 1 week and 1 month post,” no significant differences existed among the four groups.

Interestingly, as everyone faced the sudden outbreak of the pandemic, the “Stress-relief (nature) + M.E. “Group seemed to have a lesser belief that virtual nature could relieve stress at 1 week and 1 month post as compared to the “Non-stress-relief” Group and “Stress-relief (non-nature)” Group. However, their significantly lower intention to expose themselves to virtual nature for stress relief no longer existed at 1 month post, perhaps because people had no choice but to use virtual nature as a substitute for nature experiences as the pandemic continued. Group differences in attitude were present, but there were no differences in the actual frequencies of virtual nature exposure among the groups.

#### Group effect on recalls of “Positive Nature,” “Positive,” and “Nature” images

This set of questions also was added after the outbreak of the pandemic, so only 1 week and 1 month post data are available. One way between-subjects ANOVA (see Table 5) showed the “Number of recall on nature image” was not significantly different among the four groups. “Number of recall on positive image” was not significantly different for the four groups at 1 week post, but it became significant at 1 month post, at which time “Stress-relief (nature) + M.E.” Group (1 month post: *M* = 1.65, SD = 0.65) had a higher number of the recalls than “Stress-relief (nature)” Group (1 month post: *M* = 1.0, SD = 0.80). On the other hand, for “Number of recall on positive image of nature” 1 week post, “Stress-relief (nature) + M.E.” Group (1 week post: *M* = 1.39, SD = 0.99) had a higher number of recalls than “Non-stress-relief” Group (1 week post: *M* = 0.61, SD = 0.72). At 1 month post, “Stress-relief (nature) + M.E.” Group (1 month post: *M* = 1.26, SD = 0.81) had a higher number of recalls than “Non-stress-relief” Group (1 month post: *M* = 0.61, SD = 0.72) and “Stress-relief (nature)” Group (1 month post: *M* = 0.61, SD = 0.72).

The results indicate answering the motivational enhancement questions in addition to reading the motivational message related to nature resulted in participants having a significantly greater recall of positive images or positive nature images at different times as compared to the control groups. Also, “Stress-relief (non-nature)” Group may have been influenced by the positive thinking presented in their message to look for nature or positive images, resulting in this group having no significant difference with “Stress-relief (nature) + M.E.” Group in the number of recalls of positive images or positive nature images.

#### The effects of different messages on wellbeing and connectedness to nature

**FIGURE 4:**
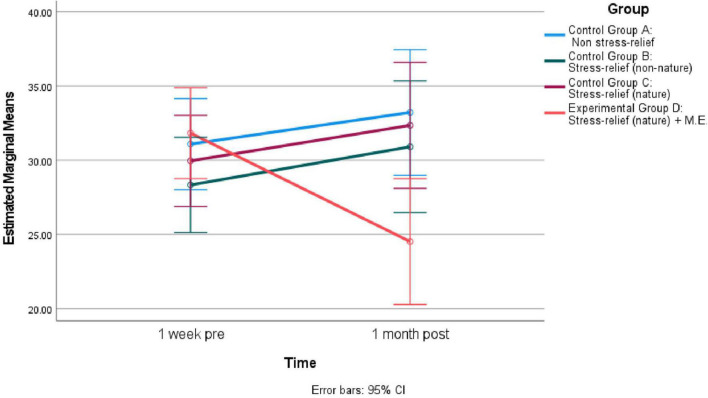
Estimated marginal means of “Perceived Stress Scale-14” across different time for four groups (*N* = 90).

ANOVA shows no significant differences in the means of the four groups at 1 week pre (see Table 5). One month later, the differences in the means of the four groups were significant. *Post hoc* comparisons using the Turkey HSD test found the mean score for “Stress-relief (nature) + M.E.” Group (*M* = 24.52, SD = 12.79) was significantly lower than “Non-stress-relief” Group (*M* = 33.22 SD = 7.66). As indicated by Figure 4, the PSS score for all groups increased from 1 week post to 1 month post, except for “Stress-relief (nature) + M.E.” Group, which experienced a decrease.

ANOVA (see Table 5) also shows for DASS_Stress, DASS_Anxiety, DASS_Depress, and Flourishing, there was not a time or time × group effect. Apparently, the experimental conditions impacted the feelings and thoughts that are directly measured by PSS-14 (65) but not the clinically significant perceived severity of symptoms related to depression, anxiety, and stress as measured by DASS-21 (69). There was no impact on the variable “Flourishing,” which relates more closely to “meaning and purpose” in life (77) and eudaimonic wellbeing (90).

**FIGURE 5:**
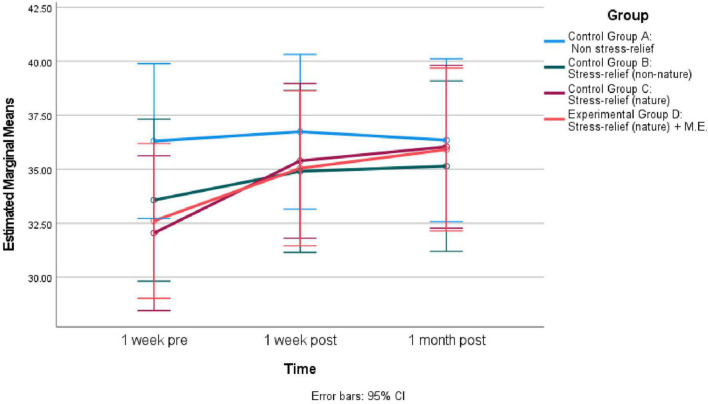
Estimated marginal means of “Connectedness to nature” across different time for four groups (*N* = 90).

The main effect of time for CNS was significant *F*(2, 172) = 9.54, *p* < 0.01, Eta2 = 0.10. Pairwise comparison showed the mean difference 1 week post vs. pre-test was 1.99 (SE = 0.51, *p* < 0.01) while 1 month post over pre-test was 2.23 (SE = 0.48, *p* < 0.01). This indicates people sought closeness with nature during the pandemic, as their connectedness to nature increased significantly regardless of their group membership. See Figure 5 for Estimated Marginal Means of CNS for the four groups across pre-experiment, 1 week post, and 1 month post.

### Research question 2: To what extent motivation indicators contributed to connectedness to nature during the pandemic

Table 6 lists the summary of Hierarchical Multiple Regression results for CNS. We interpreted the adjusted *R*^2^ instead of *R*^2^ for the adjustment in the number of predictors.

**TABLE 6:** Summary of Hierarchical Multiple Regression result on connectedness to nature (*N* = 90).

DV = Connectedness to nature										

	Pre			1 week post			1 month post			
			
Variable	*B*	SE	β	*B*	SE	β	*B*		SE	β
**Step 1:**										
Gender	−4.35	1.85	−0.25*	−3.72	1.84	−0.21	−3.85		1.93	−0.21
Age	0.54	0.37	0.15	0.41	0.37	0.12	0.46		0.39	0.12
Adjusted *R*^2^	0.06			0.03			0.03			
ΔR^2^	0.08*			0.06			0.06			
**Step 2:**										
Gender	−3.34	1.76	−0.19	−3.29	1.86	−0.19	−4.20		1.72	−0.23*
Age	0.62	0.35	0.17	0.09	0.39	0.03	−0.02		0.35	−0.01
Belief in nature can relieve stress	0.10	1.17	0.01	0.60	0.74	0.10	2.48		0.97	0.30*
Intend to expose oneself to nature for stress-relief	0.94	0.83	0.14	0.77	0.75	0.13	−0.09		0.96	−0.01
Frequency of exposure to nature for at least 20 min in last week on one’s own	2.07	0.90	0.23*	−0.44	0.98	−0.05	−0.72		1.14	−0.07
Frequency of exposure to nature for at least 20 min in last week with family or friends	2.28	1.02	0.23*	1.46	1.07	0.16	−1.04		1.96	−0.05
Belief in “virtual” nature can relieve stress	−	−	−	0.36	0.90	0.05	0.23		0.79	0.03
Intend to be exposed to “virtual” nature for stress relief	−	−	−	1.59	0.81	0.26	1.47		0.71	0.25*
Frequency of exposure to “virtual” nature for at least 20 min in last week	−	−	−	−0.63	1.04	−0.07	1.01		0.84	0.12
Number of recall on positive image related to nature in last week				1.00	1.11	0.11	3.53		1.13	0.32**
Adjusted *R*^2^	0.19			0.10					0.30	
ΔR^2^	0.17**			0.15				0.33**		

*p < 0.05; **p < 0.01.

The results of the first block of hierarchical linear regression, measuring the time before the outbreak of the pandemic, yielded a statistically significant model (*p* < 0.05) that explained 8% of the variance in CNS, namely from the control variables of gender and age. The second block analysis, too, yielded a significant model (*p* < 0.01), with “Frequency of exposure to nature for at least 20 min in last week on one’s own” (β = 0.23, *p* < 0.05) and “Frequency of exposure to nature for at least 20 min in last week with family and friends” (β = 0.23, *p* < 0.0105) significantly explaining the model and contributing 17% of variance in CNS.

At 1 week post, when the pandemic was just beginning, neither the control nor the independent variables examined were significant in the model. That means the model did not explain the variance in CNS well. This is likely because people were adjusting to the pandemic and, as a result, the usual profile of connectedness to nature was disturbed.

For 1 month post, when the pandemic has been occurring 3 to 5 weeks, the second block of variables, including belief, intention, and frequency of exposure, rendered the model significant. The *R*^2^ change indicates 33% of the variation in CNS can be explained by the variables, including gender (β = **−**0.22, *p* < 0.05), “Belief in nature can relieve stress” (β = 0.30, *p* < 0.05), “Intend to expose oneself to virtual nature for stress-relief” (β = 0.25, *p* < 0.05), and “Number of recall on positive image of nature” (β = 0.32, *p* < 0.01). When connectedness to nature was taken away by the pandemics, people pursued other methods to maintain their nature connection, and this included by cognitive means of believing nature can relieve stress, emotional means of intending to expose oneself to virtual nature, and, most importantly, by paying closer attention to positive images of nature present in daily life, as measured by the item “recall of positive image of nature,” which was the most significant variable in the model.

### Research question 3: To what extent connectedness to nature contributes to perceived stress at 3 to 5 week post blooming of the pandemic

Table 7 displays the summary of the Hierarchical Multiple Regression which examines the role of Connectedness to Nature in explaining PSS. The controlled variables namely gender, age, group membership, pre-score of PSS and pre-score of MSPSS accounted for 42% of the model’s variance. CNS was added as the block 2 variable and explained 4% of the variance of PSS (β = **−**0.20, *p* < 0.05). The negative relationship between the two variables implies increases in Connectedness to Nature are associated with decreases in Perceived Stress. Nevertheless, by comparing standardized coefficient Betas, it was revealed Connectedness to Nature contributed much less to the model than did the pre-score of PSS (β = 0.49, *p* < 0.01) and membership in the experimental groups (β = **−**0.36, *p* < 0.01).

**TABLE 7:** Summary of Hierarchical Multiple Regression result on Perceived Stress Scale-14 (*N* = 90).

	DV = Perceived Stress Scale-14		
	
	*B*	SE	β
**Step 1:**			
Gender	−0.72	1.98	−0.03
Age	−0.25	0.38	−0.06
Perceived support measured at 1 month	−0.12	0.08	−0.13
Control Group A	0.43	2.52	0.02
Control Group B	0.42	2.64	0.02
Control Group C	−	−	−
Experimental Group D	−8.53	2.56	−0.35**
PSS_pre	0.74	0.13	0.51**
Adjusted *R*^2^	0.37		
ΔR^2^	0.42**		
**Step 2:**			
Gender	−1.41	1.95	−0.06
Age	−1.15	0.38	−0.03
Perceived support_1 month	−0.09	0.08	−0.10
Control Group A	0.56	2.46	0.02
Control Group B	0.18	2.58	0.01
Control Group C	−	−	−
Experimental Group D	−8.66	2.50	−0.36**
PSS_pre	0.70	0.13	0.49**
**Connectedness to Nature measured at 1 month**	−0.23	0.10	−0.20*
Adjusted *R*^2^	0.40		
ΔR^2^	0.04*		

Remarks: Control Group C has been excluded by the Regression.

Control Group A: Non-stress-relief. Control Group B: Stress-relief (non-nature).

Control Group C: Stress-relief (nature).

Experimental Group D: Stress-relief (nature) + M.E.

*p < 0.05; **p < 0.01.

## Discussion

### General discussion

This was a randomized control trial to explore whether messages with motivational elements can lead young adults to nature exposure for stress reduction. Compared with “Non-stress-relief” Group and “Stress-relief (non-nature)” Group, participants who read the motivational message and answered motivational enhancement questions had higher levels of motivation, as indicated by their greater belief that nature can relieve stress and their greater intention to expose themselves to nature for stress relief. Such a significant difference, though it only lasted for a duration of 1 week, was not present between “Stress-relief (nature)” Group (i.e., without answering motivational enhancement questions) and the aforementioned two control groups. Also, “Stress-relief (nature) + M.E.” Group was seemingly more resistant to virtual nature, which is supported by them being less likely to believe in beneficial effects of virtual nature and less likely to intend to expose themselves to virtual nature. While frequency of exposure to nature may have been impacted by the pandemic and therefore not only in response to the experiment, a relatively higher number of recalls of positive images of nature, an indicator of behavioral change we constructed in the later part of the study, was present in “Stress-relief (nature) + M.E.” Group. The present study found a trend of increasing amounts of perceived stress across the three control groups, which aligns with the relationship for young adults between stress and the COVID-19 pandemic (5–8). Interestingly, there was a trend of decreasing perceived stress for “Stress-relief (nature) + M.E.” Group.

These favorable findings for motivational indicators, together with stress reduction effects, reflect the success of the motivational enhancement strategy. The strategy supports the notion that, to promote help-seeking in young people, the role of the internet and online resources should be treated as an adjunct to offline help-seeking (91). Our design of the motivational message aligned with the self-determination theory as proposed by Ryan and Deci (24, 25), that is, supporting autonomy to visit nature, enhancing self-efficacy by suggesting an easily accomplished task (i.e., simply expose yourself to nature), and addressing connection with the larger community including nature. However, in the present study the message solely addressing the said concepts did not increase motivation of the young adults to expose themselves to nature. It was only with ambivalence addressed as is recommended by the Motivational Enhancement Therapy, or Motivational Interviewing (31), that motivation of young adults for nature exposure increased. Young adults, a self-reliant population that enjoys informal help-seeking (92), perhaps are more receptive to an indirect approach that grants them freedom for ambivalence resolution. By asking the young adults to assume a third person view in solving problems they may also encounter, we successfully addressed their resistance to an extent. Defining the nature experience as not restricted to a physical visit to nature, as suggested by Hunter et al. (18), may also contribute to a pro-attitude of nature exposure in young adults.

Very often physical exposure to nature is regarded as a behavioral indicator for motivation of nature exposure. Our study indicates that, apart from actual physical exposure, recalls of positive nature elements can also be increased by motivational enhancement work. The recall somehow reflects the corresponding attention to the positive nature stimuli. Though it did not attain a stress reduction outcome as Hunter (18) yielded for 20 to 30 min subjective nature experiences, its impact on connectedness to nature can results in positive wellbeing outcomes, including changes in perceived stress, depression, anxiety and flourishing (81). This finding implies behavioral measurement of exposure to nature may be shorter or less deliberate, as we expected, or it can present as attention to positive stimuli in nature.

Shortly after all participants in this study completed the 1 week pre-test, the outbreak of the pandemic occurred in Taiwan. We had the opportunity to examine how motivational indicators could explain connectedness to nature, as well as whether perceived stress could be explained by connectedness to nature, a variable that is associated with human wellbeing (81, 82). The contributing variables differed during different phases of the pandemic, with actual physical contact, typically a contributing variable to nature connectedness, vanishing once the outbreak began. Gradually, as people adjusted to the pandemic, their belief in physical nature’s ability to heal, their intention to connect with virtual nature (while physical contact was still hindered), and recall of positive images of nature contributed to their connectedness to nature. This implies humans are flexible in adopting different means to attain closeness to nature when the actual environment hinders it.

Thus far, empirical studies of connectedness to nature have mainly centered on closeness with actual forests [e.g., (93)] and urban greenspaces [e.g., (94)]. The present study echoes findings of previous studies, that virtual or simulated nature (95, 96) can be one source of human connectedness to nature, as we found that people who intended to expose themselves to virtual nature had a higher connectedness with nature. Although the effectiveness of virtual nature in increasing positive moods is inferior to outdoor exposure (97), it remains a possible substitute for nature among young adults, especially when actual access to nature is deprived, as was the case during the pandemic.

For young adults stress is associated with different life domains. Li et al. (8) reported a significant inverse relationship between perceived social support and psychological symptoms during the COVID-19 pandemic. However, by comparing the coefficient of beta in the regression, our study surprisingly found the influence of connectedness with nature on perceived stress is double that of perceived social support. We can say promoting connectedness to nature during a health crisis like the pandemic would be beneficial to the wellbeing of young adults and such benefits may be greater than those from their perceived social support.

Connectedness to nature, a fluid quality reflecting how much a person feels emotionally connected to the natural world (80), increased across the four experimental groups over time during the pandemic. Although there was not a treatment-free group in the present study for comparison, we believe the increased connectedness to nature is a result of the pandemic rather than the experiments, given that logically it is unlikely being subjected to a message about online shopping security (as was the case for Control Group A) would lead to increased connectedness with nature. Humans possess an innate tendency to seek connection with nature (98) and they hunt for “atmospheres of safety and belonging’ [(99) p. 68]. While the pandemic affected the entire world, some researchers have attributed this health crisis to global human-nature interactions (100) and have advocated for the protection, restoration, and promotion of sustainable use of terrestrial ecosystems for preventing future pandemics (101). Such literature has led individuals to reexamine the human-nature relationship and perhaps raise their tendency to have closeness with nature. Our present study found subjecting individuals to motivational messages did not help increase connectedness to nature. Exploration of other methods to promote nature connection should be a future research item.

While we conducted an online research study, we had to be extra cautious during the study administration. Because the number of survey items can affect the drop out of an online study (102), we controlled the number of questions across every stage of the experiment to below 100 questions. To further decrease drop out, we politely reminded participants when the deadline for questionnaire completion was approaching. This resulted in a very low drop out (i.e., only three dropouts) across all stages of the study. Also, we detected potential response set by examining the questionnaire immediately, and we allowed participants to change their answers, with the hope of raising the reliability of the study. Most importantly, we responded promptly when the pandemic began and innovatively added measurements that potentially could provide meaningful information about the relationship during the pandemic between humans and nature. Finally, we made the remuneration minimal, $400 Taiwanese dollars, to not confound the motivation of participants. By applying our understanding of the characteristics of online studies and young adults and the pandemic’s impact on human behavior, we hope we can maximize the rigor of the study.

### Limitations

There are a number of limitations of the study. First, we note the time frame of the study relatively coincided with the outbreak of the COVID-19 pandemic. However, because the questionnaires asked the participants to refer to the past week, during the 1 week post responses there may be participants who referred to conditions before the city locked down, rendering the 1 week post findings a mixture of responses. Also, the opening of the message read by Groups B, C, and D was not adjusted to address stress relating to the pandemic. We must admit these slight incongruences between the study materials and timing of the study are things we could not control in a dynamic situation, and they may have affected the experimental effects.

We notice the participants’ accessibility to urban Greenland and the surrounding environment (e.g., congestion, urban constructions) can be varied according to their residence. These are the variables that the present study had not inquired as Taiwanese students used to travel among school, dormitories and their home thus the accessibility to Greenland is difficult to be defined. Or if rigidly defining it the creation of bias would be very likely. We finally decide to let the random sampling to even out the influence of this variable.

Single-item questions for motivation, including belief, intention, and frequency, hindered the investigation of psychometric properties such as reliability. Although clustering of the same items across different time periods in correlations could serve as evidence of the items’ validities, further construction of multi-dimensional motivational indicators for each construct is warranted. Similarly, we added questions addressing virtual nature and recall of impressive images from daily life peremptorily to supplement information of human relationships with nature during the pandemic. Though meaningful findings were yielded for these variables, further validation of these measurements is required.

The total of 90 study participants guaranteed power of the statistical analyses, but it still imposed limitations for conducting mediation analysis on variables, including four group membership, motivational index, outcome measurement on wellbeing, and controlled variables such as age, gender, and perceived social support. For the sake of upholding a certain power of the tests, we could only conduct separate hierarchical regressions to examine the relationships among connectedness to nature, the motivational index, and the outcome measurements. Fortunately, through this approach we managed to generate meaningful findings for understanding the subject matter.

Constrained by the number of participants, we eliminated a control group involving motivational enhancement questions only (i.e., without reading the motivational message). With this additional control group, we would have examined the pure effect of the motivational enhancement questions. Future study is warranted to include this control group so that clarity may be attained regarding what constitutes an effective motivational message or strategy in mobilizing people to connect with nature.

### Conclusion

Literature review indicates the increasing stress experienced by young adults should not be neglected during the pandemic, nature offers potential benefits on wellbeing, and young adults are more receptive to intervention approaches that address their need for autonomy. By addressing ambivalence, as is suggested by the motivational enhancement approach, in addition to core principles of motivation, we induced young adults to display favorable changes in motivational indicators in terms of belief and intention to connect with nature to relieve stress. In this intervention the young adults exhibited greater recall of positive nature memories from their daily lives and reported lower perceived stress 1 month after the experiment. Our study explored indicators measuring motivation for nature exposure during the pandemic when real exposure to nature was less likely. During the pandemic and after, people have been more health conscious and seemingly more likely to connect with nature, so it may be a golden time to consolidate such motivation to benefit their physical and mental health.

## Data availability statement

The datasets presented in this study can be found in online repositories. The names of the repository/repositories and accession number(s) can be found below: https://doi.org/10.6084/m9.figshare.19608258.v1.

## Ethics statement

The studies involving human participants were reviewed and approved by Research Ethics Committee of National Taiwan University. The patients/participants provided their written informed consent to participate in this study.

## Author contributions

Y-YY conceived and designed the program, collected the data, wrote and amended the manuscript. C-PY gave advice on the program design, reviewed the manuscript and acquired funding for the study. Both authors read and agreed to the published version of the manuscript.

## Appendix

A. Measurements on Motivation for Nature Visit.

Please circle number on the scales and fill in the blanks.

1. “Exposure to nature can relieve stress.” To what degree do you agree with this statement?

Answer on seven point Likert Scale: 1: totally disagree; 2: Some degree of agreement and some degree of disagreement; 7: totally agree.

2. “To what degree do you intend to expose yourself to nature for stress relief?”

3. “In the last week, how many times have you exposed yourself to nature alone for at least 20 min?”

Answer: _______times.

4. “In the last week, how many times you have exposed yourself to nature with family and friends for at least 20 min?”

Answer: _______times.

5. “Exposure to virtual nature can relieve stress.” To what degree do you agree with this statement?

6. “To what degree do you intend to expose yourself to nature for stress relief?”

7. “In the past week, how many times have you exposed yourself to virtual nature for at least 20 min?”

Answer: _______times.

Recall of three impressive images in the last week

“Our eyes are comparable to a camera lens while our heads are comparable to hard disks of our memory. Whether these images are from indoors or outdoors, they can elicit feelings. Please recall images from your daily living that brought you feelings in the past week. Write three of these images, describe them in detail, and then determine whether they brought you positive, negative, or mixed/uncertain feelings.”

B. Image 1: _______________________________________________________________________________________________

Classify it as (please tick one only):

• Positive

• Negative

• mixed/uncertain feelings

Image 2: _______________________________________________________________________________________________

Classify it as (please tick one only):

▢ Positive

▢ Negative

▢ mixed/uncertain feelings

Image 3: _______________________________________________________________________________________________

Classify it as (please tick one only):

▢ Positive

▢ Negative

▢ mixed/uncertain feelings
